# Identification and Classification of Snack-Type Watermelon (*Citrullus lanatus*) Genotypes Using Seed Morphology and Machine Learning Techniques

**DOI:** 10.3390/foods14234069

**Published:** 2025-11-27

**Authors:** Uğur Ercan, Sıtkı Ermiş, Onder Kabas, Güleda Öktem, Aylin Kabas, Gigel Paraschiv

**Affiliations:** 1 Department of Informatics, Akdeniz University, 07070 Antalya, Türkiye; ugurercan@akdeniz.edu.tr; 2 Department of Horticulture, Faculty of Agriculture, Eskişehir Osmangazi University, 26040 Eskişehir, Türkiye; ermis@ogu.edu.tr; 3 Department of Machine, Technical Science Vocational School, Akdeniz University, 07070 Antalya, Türkiye; okabas@akdeniz.edu.tr; 4 Variety Registration and Seed Certification Center, 06172 Ankara, Türkiye; guledaoktem@gmail.com; 5 Department of Organic Farming, Manavgat Vocational School, Akdeniz University, 07070 Antalya, Türkiye; 6 Department of Biotechnical Engineering, Faculty of Biotechnical Engineering, National University of Science and Technology Politehnica Bucharest, 060042 Bucharest, Romania

**Keywords:** watermelon, classification, ROC-AUC score, random forest

## Abstract

This study examines the effectiveness of machine learning approaches for the automatic identification of watermelon genotypes from the seeds of watermelon, for the snack-type watermelon (*Citrullus lanatus*). Nine genotypes with red, white, and black seed coats were assessed in total. For each genotype, 200 seeds were analyzed using high-resolution imaging and digital measurement techniques for the extraction of morphological characteristics (length, width, thickness, area, perimeter, equivalent diameter, etc., and physical (weight) and colorimetric attributes of the (L, a, b). The resulting dataset was modeled using Artificial Neural Network (ANN), Random Forest (RF) and Extra Tree (ET) algorithms and performance was validated by a 10-fold cross-validation. The primary objective of the study was to match (identify) each seed accurately with its respective genotype by using the morphological, physical, and colorimetric characteristics of the seed and thus to perform genotypic classification. The comparative results showed that the RF model had the highest genotypic performance (accuracy 92.22%, F1-score 91.87%, Cohen’s Kappa 0.9118), followed by the ET (accuracy, 90.00%) and ANN models with a relatively lower precision (86.11%). Statistical analysis using the Wilcoxon signed-rank test confirmed that both RF and ET significantly outperformed ANN, with RF providing superior balance and stability over ET. The findings highlight that machine learning-based frameworks enable rapid, reliable, and non-destructive classification (identification) of snack-type watermelon seeds according to their genotypes. Such approaches hold strong potential for enhancing varietal traceability in breeding programs, improving quality control in commercial seed production, and meeting the high-throughput demands of seed processing industries.

## 1. Introduction

Watermelon (*Citrullus lanatus*) is cultivated extensively worldwide and is widely valued for its flavor and significant water content. While the succulent flesh is predominantly consumed fresh, the seeds are often roasted and consumed as snacks or processed for oil extraction [1]. Particularly those from edible, snack-type varieties have maintained culinary and cultural importance across various regions for generations. In countries such as India, parts of Africa, and several areas in the Middle East and Asia, watermelon seeds are traditionally consumed as salted and roasted snacks, in addition to their use in oil production [2,3]. Recently, these seeds have garnered increased attention for their applications in modern food systems, including in gluten-free formulations and as fermented flavor enhancers [4]. Additionally, research has investigated their role as natural coagulating agents in water treatment processes [5]. In Türkiye, particularly in the Southeastern Anatolia region, specific watermelon genotypes are cultivated to satisfy regional preferences, with a particular focus on seed size, test a texture, and flavor characteristics suited for traditional consumption practices. Watermelon seeds have been consumed as a snack in this region for decades, typically following roasting or boiling [6].

Beyond their cultural significance, snack-type watermelon seeds possess substantial nutritional value. They are characterized by a high content of plant-based protein (17%), dietary fiber (42%), carbohydrates (12%), and lipids (26.5–27.8%), in addition to essential micronutrients such as calcium, phosphorus, potassium, magnesium, sodium, and zinc [7]. Furthermore, these seeds are notable for their significant levels of phytochemicals and exhibit strong antioxidant activity, making them promising candidates for functional food applications [8]. With the growing global emphasis on sustainable, plant-based, and health-conscious nutrition, these seeds are gaining increased attention from both consumers and the food industry.

Traditionally, seed classification has relied on visual inspection and basic morphological characteristics. However, these methodologies often prove insufficient for distinguishing between closely related watermelon genotypes, especially when phenotypic variations in seed size, shape, or surface texture are subtle or overlapping. Optical sorting systems and binocular microscopy, even when executed by skilled operators, frequently yield classification accuracies of only 70–80% for morphologically similar varieties [9]. This limitation poses significant challenges for breeding programs and commercial seed production, where precision and consistency are paramount. Consequently, machine learning (ML) techniques have emerged as a robust alternative, offering high-throughput, objective, and scalable solutions [10]. By identifying intricate patterns in seed morphology that may escape human observation, ML models considerably enhance the reliability and efficiency of genotype classification.

Recent advancements in machine learning (ML) and computer vision have transformed seed classification from a manual, subjective process into an automated, data-driven approach. Research on various crops including dry beans (*Phaseolus vulgaris* L.) [11], soybean (*Glycine max* L.) [12], maize (*Zea mays* L.) [13], and ornamental pumpkin seeds (*Cucurbita pepo* L. var. *ovifera*) [14] has demonstrated that Convolutional Neural Networks (CNNs) and classical ML algorithms such as Support Vector Machines (SVM), Random Forest (RF), Logistic Regression (LR), and k-Nearest Neighbors (kNN) can achieve classification accuracies exceeding 85–95% by utilizing morphological, color, and texture features extracted from seed images. These methodologies detect subtle phenotypical differences that are imperceptible to human evaluation and facilitate non-destructive, rapid, and repeatable quality control in commercial seed production. Specifically, in watermelon seed phenotyping, X-ray imaging combined with ML has achieved accuracies of up to 87.3% using ResNet-50 [15], while near-infrared hyperspectral imaging (NIR-HSI) integrated with PLS-DA models has attained viability discrimination rates ranging from 77.8% to 91.8%. Furthermore, ensemble deep learning models that incorporate architectures such as LeNet, ResNet, and GoogLeNet have reached classification accuracies of up to 87.97% [16]. The analysis of the scientific literature by one of the most recent articles [17] highlights that a combination of high-throughput phenotyping and deep learning is a promising direction for scalable, non-destructive seed quality evaluation, which supports the feasibility of the technologies to the classification of snack-type watermelon genotypes. To this extent, some of the recent papers have already proven that deep learning-based methods can be efficient in analyzing agricultural seeds. The results of a CNN-based deep learning model with 100 percent accuracy in classifying rice and non-rice crops and 0 percent precision, recall, and F-score in the non-rice class were very good because of the imbalance between the labels [18]. They discovered that with the lightweight soybean seed defect identification network (SSDINet) utilizing the seed-based contour detection (SCD) preprocessing algorithm, the model could achieve 98.64% accuracy only using 1.15 million parameters within 4.70 milliseconds [19]. In four simple seed analysis problems, namely damaged seed detection (DaS), diseased seed identification (DiS), single-crop seed classification (SCS), and different-crop seed classification (DCS), Kumar et al. [20] investigated the employment of the Machine Learning (ML) and Deep Learning (DL) methods. They discovered that with up to 99.73 (neuro-fuzzy) and 96.00 (discriminant analysis) accuracies, ML approaches, typically built on the basis of RGB data, and DL approaches, which increasingly relied on hyperspectral imaging, had higher accuracy rates, such as 99 percent with transfer-learned VGG16, 100 percent with maize varieties, and 99.80 percent accuracy with ANN + SqueezeNet features. Rajalakshmi et al. [21] developed a new ViT-based deep neural network model called RiceSeedNet which classifies 13 local rice seed varieties. They compared it to traditional CNN-based methods and found it to be better, with 97% accuracy on a private RiceSeed dataset and 99% accuracy on a public dataset which consists of eight different varieties of rice and achieves 99% accuracy on eight different varieties of rice and 99% accuracy on a public dataset. They found it to be better than traditional CNN-based methods.

In this study, we investigated the classification of nine snack-type watermelon (*Citrullus lanatus*) genotypes utilizing machine learning techniques. By integrating morphological descriptors with advanced classification algorithms, we aimed to develop a non-destructive, scalable, and highly accurate approach to facilitate genotype differentiation in commercial seed production. The proposed framework enhances varietal traceability and selection within breeding programs, and provides a practical and transferable solution for industrial seed quality control, thereby bridging the gap between traditional practices and advanced machine learning applications in horticultural crops. Accordingly, the key contributions and distinctive aspects of this study to the existing literature and scientific knowledge are outlined below.

• The genotyping of nine watermelon genotypes of the seed stock based on red, white, and black seed coats was performed using the morphological, physical, and colorimetric (L, a, b) characteristics of the seeds.
• ANN, Random Forest (RF), and Extra Trees (ET) algorithms were relatively compared, and it was shown that the best performance was achieved by the RF model with an accuracy of 92.22 and a Cohen Kappa value of 0.9118.
• The Wilcoxon signed-rank test statistically proved that the RF and ET models were significantly superior to the ANN model, which demonstrates the high stability of the ensemble-based approaches.
• The suggested ML-based framework can be used to offer a fast, accurate, and non-destructive classification framework able to fulfill the high-throughput needs of the seed processing industry, the breeding programs enhancing varietal traceability, and the quality regulation in commercial seed production.
• The research paper helps to fill the gap between traditional methods and more modern machine learning applications providing concrete evidence of the relevance of computer vision and artificial intelligence, especially in the domain of horticultural seed science.

## 2. Materials and Methods

### 2.1. Plant Material and Growth Conditions

The study was carried out in the Beydere Seed Certification Testing Directorate, located in the Manisa Province district of western Türkiye (38°43′55″ N, 27°30′36″ E), approximately 40 m above sea level. This site is located within the inner Aegean region and is characterized by a typical Mediterranean climate, which features hot, dry summers and mild, rainy winter conditions that are conducive to watermelon cultivation. To assess the soil properties of the experimental site, composite samples were collected from the 0–30 cm soil depth and analyzed at a certified soil laboratory. The results indicated that the soil was slightly alkaline (pH 8.00), non-saline (637 µS/cm), and classified as clay-heavy (49% field capacity), with a low organic matter content (0.70%) and moderate lime concentrations (4.68%). Furthermore, the soil was categorized as deficient in organic matter, low in available phosphorus (6.64 ppm), and rich in available potassium (238 ppm).

**Figure 1:**
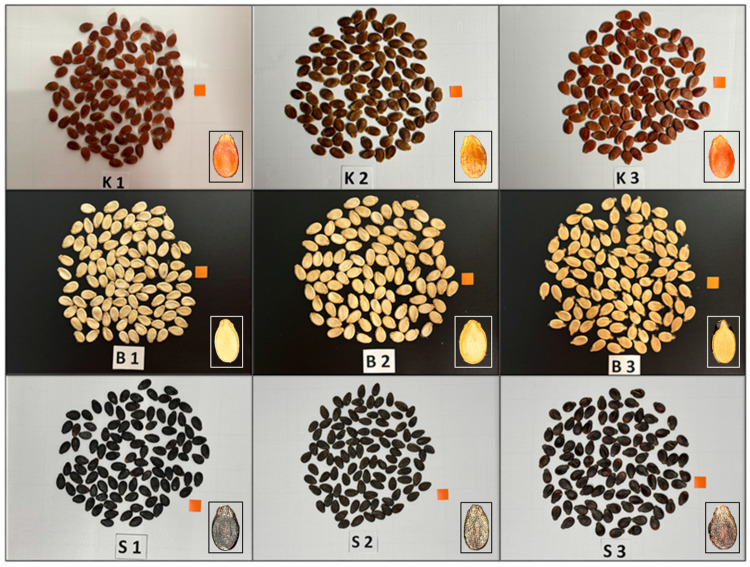
Representative seeds from nine snack-type watermelon (*Citrullus lanatus*) genotypes utilized in the study are illustrated.

A total of nine snack-type watermelon (*Citrullus lanatus*) genotypes were evaluated in this study, each selected based on their distinct seed coat coloration. The genotypes were evenly distributed across three color groups, with three genotypes per group. Specifically, GSB-1-22, GSB-2-22, and GSB-11-22 exhibited black seed coats; GSW-87-22, GSW-17-22, and GSW-47-22 displayed white seed coats; and GSR-49-22, GSR-71-22, and GSR-113-22 were characterized by red seed coats. These seed color classifications, along with other morphological seed characteristics, were employed as key parameters in the machine learning framework established for genotype differentiation. Representative seed images of each genotype, categorized by seed coat color, are presented in Figure 1.

The top row features genotypes with red seed coats (GSR-49-22 (K1), GSR-71-22 (K2), GSR-113-22 (K3)), the middle row represents those with white seed coats (GSW-87-22 (B1), GSW-17-22 (B2), GSW-47-22 (B3)), and the bottom row displays genotypes characterized by black seed coats (GSB-1-22 (S1), GSB-2-22 (S2), GSB-11-22 (S3)). Each label, consisting of a letter and number, denotes a specific genotype. Detailed information on the genotypes and their seed coat colors are summarized in Table 1.

The field experiment was structured in a Randomized Complete Block Design (RCBD) with two replications. Each block contained all nine genotypes, which were randomly assigned to individual plots. Each plot consisted of a single row with ten plants, established at a spacing of 1.5 m between rows and 0.8 m between plants within the row. Fruits were harvested upon reaching physiological maturity, as indicated by the leaf drying and desiccation of the fruit peduncle, which facilitated easy detachment from the stem. Only fruits resulting from self-pollination were harvested, and each fruit was distinctly labeled. Following harvest, the fruits were cut open, and the seeds were carefully extracted. The seeds were subsequently dried in a controlled environment chamber set at 25 °C and 35% relative humidity for 72 h to ensure uniform moisture content prior to further processing.

### 2.2. Data Collection Site

The morphological characteristics of the watermelon seeds were determined using digital image analysis. High-resolution images of the seeds were obtained using a Canon EOS 80D digital camera (Canon Inc., Tokyo, Japan) with a standard macro lens. Image analysis was performed using the open-source ImageJ software (version 1.53, National Institutes of Health, Bethesda, MD, USA).

Image Calibration: The image was spatially calibrated prior to the analysis so as to find the conversion factor between the units in a pixel and the metric dimensions (in millimeters). One of the reference objects with known physical dimensions was put in the image field and its dimensions recorded in pixel coordinates and in the units of magnitude (mm). The scale factor (conversion ratio between pixels and millimeters) has been determined using the formula below: scale factor = known distance (mm) + measured distance (pixels). All the further measurements were then converted to absolute units of measure by applying this conversion factor to all the measurements. Image processing and thresholding image preprocessing have been performed to eliminate noise background and shadow on the image of melon seeds. The black and white images are generated based on a threshold algorithm, as opposed to gray-scale. The Otsu automatic thresholding method was used, which sets the optimal threshold value by maximizing the variance between the classes (or, the reverse, minimizing the variance within classes) of the values of the pixel intensities of the foreground and the background. This algorithm considers every possible threshold value in turn and chooses the one that maximally distinguishes between the pixels of the seed (which are white and black) and the background in order to display a clear demarcation of the seed. Seed analysis: The thresholded binary images of seed analysis were automatically processed by the Combined Component Labeling (CCL) algorithm to identify each melon seed. It is a binary image scanning algorithm which labels groups of spatially connected background pixels (seed) that have a connectivity (typically four or eight connectivity zones). Morphological features were drawn out of every labeled area after labeling. The parameters that were obtained were area (sum of all the number of pixels within a given connected area), perimeter (length of boundary of each area), and other shape statistics like aspect ratio, perimeter, and size of the encasing box [14,22].

**Figure 2:**
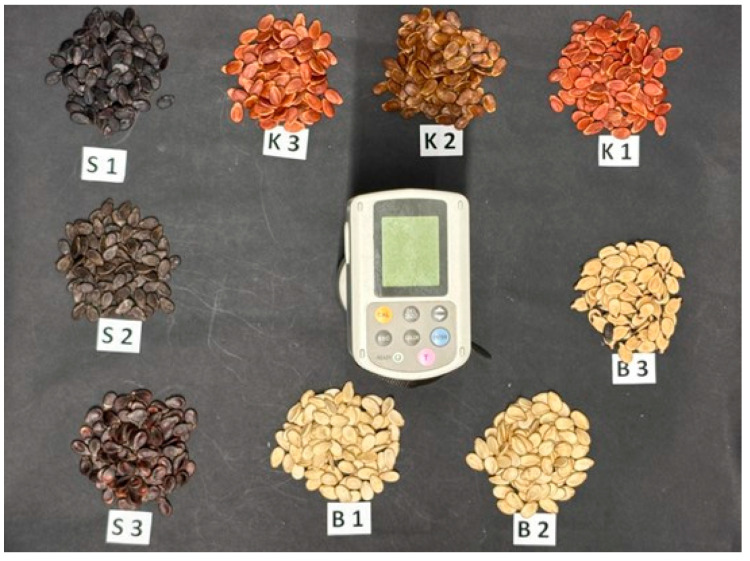
The color measurement of watermelon seeds was conducted utilizing a colorimeter.

The visible color characteristics (L, a, b) of the watermelon seed cultivars were determined using a digital colorimeter (Chroma Meter CR-400 (Konica Minolta, Tokyo, Japan)) (Figure 2) [14,23].

The mass of watermelon seeds (M, in grams) was measured using a high-precision analytical balance (Model GX-4000, A&D Company, Ltd., Tokyo, Japan), capable of measuring to an accuracy of ±0.01 g.

**Figure 3:**
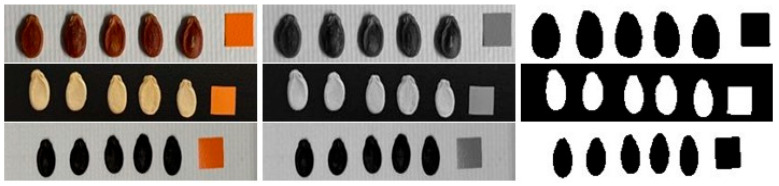
Sequential workflow and processing stages of digital images utilizing ImageJ.

The images of the watermelon seeds were taken with a custom-designed imaging box eliminating any external light interference and shadow formation. A lighting system was set to 1000 lux, and a camera was mounted perpendicularly (90°) to the box surface. The use of a stable camera stand maintained 30 cm from the camera to the seed sample to ensure consistency in the imaging geometry. The seeds were placed on a dark background to aid in image processing. The same environmental conditions and manual exposure settings were applied to each image to ensure consistency. Images of 200 randomly selected seeds from each variety were captured for image analysis. The resulting seed images were processed using the open-source software ImageJ v1.53k. The primary photographs were first converted to grayscale and then to binary (black and white) format, as shown in Figure 3.

Thirteen morphological and color-based parameters were extracted from each seed image using ImageJ and complementary measurement devices. These included length, width, thickness, mass, color components (L*, a*, b*), projected area, perimeter, length/width ratio, compactness, roundness, and equivalent diameter, forming a 13-dimensional feature vector per seed. Feature importance analysis revealed that the length/width ratio, compactness, and L* (brightness) were among the most discriminative features for classifying genotypes.

In the morphological analysis of watermelon seeds, shape factors were determined in addition to basic measurements. These factors were calculated to quantitatively describe the shape of the seeds. The morphological parameters provided by ImageJ were used for these calculations.

The height/width ratio is the ratio of the length of the kernel to its width, a factor indicating the degree of elongation of the kernel [24].

Compactness, the tightness of an object, indicates how close its shape is to a circular shape. For a perfect circle, this value is one. This value is calculated using the area and perimeter length using the following formula (1) [25]:

(1) C=P24 × π A 

where C represents the unitless compactness, A represents the area in mm^2^, and P represents the perimeter in mm.

Roundness is calculated using the area and length (major axis, L) values to indicate the amount of deviation from the ellipse shape of the seeds (2) [26]:

(2) R=4 Aπ × L2

where R represents the unitless roundness, A represents the area in mm^2^, and L represents the length in mm.

The equivalent diameter is the diameter of an imaginary circle with the same area as the analyzed seed. This parameter is used to express seed size with a single numerical value. This value is calculated using the surface area of the watermelon seed using the following Formula (3) [27]:

(3) De=2 ×Aπ

where D_e_ represents the equivalent diameter in mm and A represents the area of the watermelon seed in mm^2^.

### 2.3. Machine Learning

**Figure 4:**
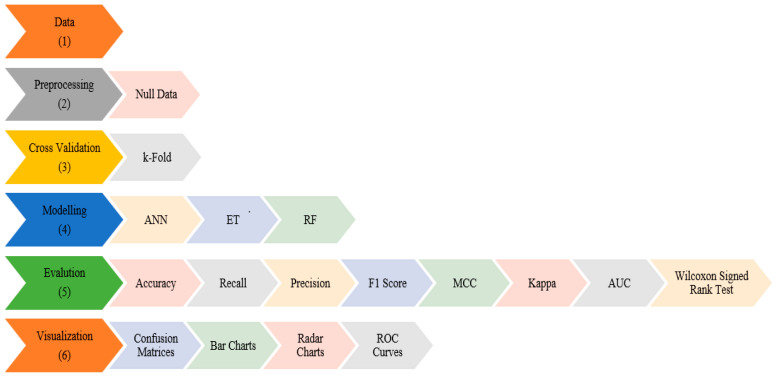
Data mining process and details of the study.

Discovering the hidden information in the database or any data store is a process [14]. This process is called data mining. Figure 4 shows the data mining process and details of the study. The process consisting of several successive steps begins with the selection of the dataset to be analyzed. The second step is the data preprocessing phase. In this step, it is investigated whether there is missing data in the dataset. There is no missing data in the dataset used. Another procedure performed during the preprocessing phase is normalization.

In the third stage, k-fold cross-validation was used. This is a validation method widely used in ML studies to use the dataset efficiently, reduce sampling variance, increase reliability, and reduce over or under-fitting [28,29]. The fold number (k) is generally used in the literature as five or ten [30]. In this study, the number of folds was taken as ten. The fourth step is the stage in which machine learning algorithms are applied. In this step, RF, ANN, and ET methods are used. These methods are successful algorithms that are widely preferred in classification studies. ANNs have become increasingly popular in agricultural classification problems due to their remarkable ability to model complex and nonlinear relationships between variables. Ensemble learning methods such as RF and ET have proven to be highly effective in agricultural classification problems due to their robustness, interpretability, and strong generalization performance. Both methods are based on aggregating the predictions of multiple decision trees to minimize overfitting and variance, leading to more stable and accurate models. In agricultural datasets, where variables are often noisy, correlated, and influenced by environmental or biological factors, RF and ET can efficiently capture complex feature interactions without requiring extensive parameter tuning [31,32,33,34,35,36]. In the fifth step, the results obtained in each fold are evaluated. accuracy, precision (macro), recall (macro), Matthew’s Correlation Coefficient (MCC), Cohen’s Kappa (Kappa), the F1-score (Macro), and ROC are metrics used in multi-class classification tasks. In the sixth step, the confusion matrices and the visualization of the metric results are realized. Then the results obtained are interpreted. This study aims to classify the snack-type watermelon seeds by means of machine learning methods using the seeds’ morphological, physical, and color characteristics.

### 2.4. Random Forests

RF is a potent ensemble learning method that is frequently applied to regression and classification problems. As the basis of the method, the principle of “Bootstrap Aggregating” and the random attribute selection principle are used. In the method, a large number of decision trees are trained in parallel, and their predictions are combined. Each tree is built on a different subset randomly selected from the original dataset, while a randomly selected attribute subset is used in each node split. This type of randomness increases the diversity between trees, reducing the model’s tendency to overfitting, while strengthening its generalizability. High accuracy, robustness, reduced susceptibility to outliers and noise, and the ability to deal effectively with large-scale datasets and high-dimensional feature spaces are the most important advantages of the model [30,37,38,39].

### 2.5. Artificial Neural Networks

ANNs, designed to process information in a parallel and distributed manner, imitate the human brain and the working structure of neurons [40]. The immense power of ANNs lies in their ability to learn from data. Through a process known as training, the network’s connection weights are iteratively adjusted to capture complex patterns within the data. A seminal algorithm driving this process is backpropagation, which efficiently adjusts the weights by propagating error signals backward through the network [41]. This training enables the network to create powerful representations of the data, allowing it to generalize and make predictions on new, unseen examples [42]. The capacity for learning complex patterns and generalizing from data has led to the widespread application of Artificial Neural Networks across a vast spectrum of scientific and industrial domains. They have proven to be exceptionally effective in solving problems that are difficult to model with traditional algorithms, particularly in areas like pattern recognition, classification, and forecasting [43]. ANNs have become increasingly popular in agricultural classification problems due to their remarkable ability to model complex and nonlinear relationships among variables. Unlike traditional statistical models, ANNs do not require strong assumptions about data distributions, which makes them particularly suitable for heterogeneous and noisy agricultural datasets [32].

### 2.6. Extra Trees

ET is one of the ensemble learning methods like RF. It is based on random variations in decision trees. The main difference is the double randomization mechanism. This involves sample selections for each tree (Bootstrap) and the completely random selection of split points. Unlike the traditional Random Forest, the best split point is not calculated; random threshold values are generated for each feature and one of them is randomly selected [44,45]. This approach reduces the variance of the model, increases computational speed, and reduces the risk of overfitting. It also gives effective results in high-dimensional data and noisy datasets. It is especially prominent in high-dimensional regression and classification problems. This random splitting strategy strengthens the generalizability of the ensemble by maximizing the diversity among the trees. It draws attention, with its lower training time compared to Random Forests and Gradient Boosting, especially in imbalanced datasets [46,47].

In ML models, parameter settings have been made to obtain the best results from the methods used. For this purpose, the results were obtained using the parameter values that yielded the best results through grid search. The parameter values for the models are given in Table 2.

## 3. Results and Discussion

### 3.1. Statistical Analysis

Table 3 displays the descriptive statistics of the dataset utilized in the study. The data were collected by the Faculty of Agriculture at Eskişehir Osmangazi University. The target variable, genotype, is categorical and consists of nine distinct classes. There are a total of 900 watermelon seeds of three different colors (red, black, and white). The minimal dataset representing 30% of the raw measurements used in this study is provided in the Supplementary Materials (Supplementary File S1). Each color is divided into three different genotypes between itself (all types and their frequencies are equal, 100). This variant takes the values GSW-087-22, GSB-001-22, GSR-113-22, GSB-002-22, GSR-049-22, GSW-017-22, GSR-071-22, GSW-047-22, and GSB-011-22. The skewness and kurtosis values of the watermelon seed data range between [−2, +2]. Accordingly, it can be said that the variables are normally distributed. The implementation of the RF, ET, and ANN models, the calculation of performance metrics and statistics, and the graphics were achieved using the scikit-learn library (version 1.7.2) [48].

### 3.2. Fold Basis Results and Interpretation

**Figure 5:**
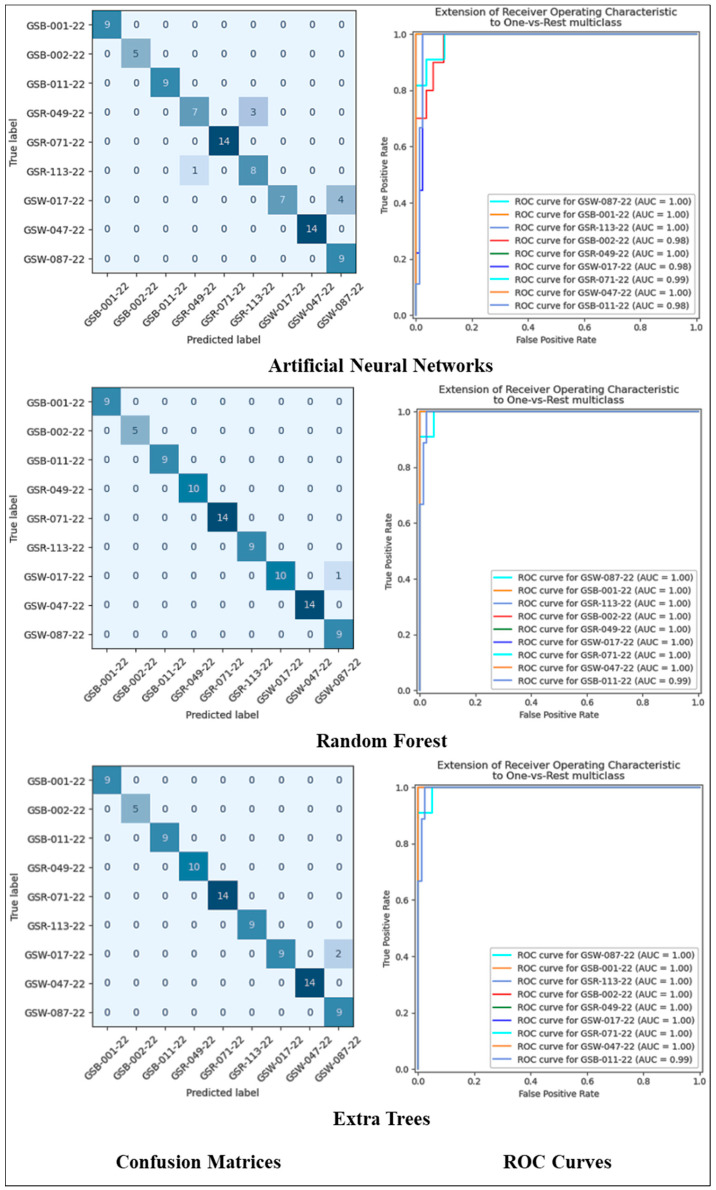
Confusion matrices and ROC curves of machine learning models in the tenth fold.

If cross-validation is used in modeling, a confusion matrix is created for each fold and then metrics are calculated using this matrix. Then, the arithmetic mean of all metrics is taken. However, due to the difficulty of displaying the confusion matrix created for each fold, only the confusion matrix and ROC of the last fold are shown and interpreted. The confusion matrices of the tenth fold are shown in Figure 5. When these matrices were interpreted, the following results were reached. All black watermelon seeds (GSB-001-22, GSB-002-22, and GSB-011-22) were correctly classified by all three classification algorithms. All red watermelon seeds (GSR-113-22, GSR-049-22, and GSR-071-22) were correctly classified by the ET and RF algorithms. In contrast, the ANN algorithm correctly classified two classes of red watermelon seeds (GSR-113-22 and GSR-071-22) but made three misclassifications when classifying one type of red watermelon seed (GSR-049-22).

White watermelon seeds labeled as GSW-047-22 and GSW-087-22 were correctly classified by all algorithms. However, while classifying the white watermelon seeds named GSW-017-22, the ANN, RF, and ET algorithms misclassified four, one, and two seeds, respectively. These comments were made only for the tenth fold.

The metric-based statistics of the folding results of the ANN, RF, and ET models are shown in Table 4.

When the “Average” values in the table are examined, it is seen that the Random Forest (RF) model performs significantly better than the other two models in all analyzed metrics. Interestingly, the ANN model has the lowest standard deviation (SD) values across all metrics. This shows that ANN is the most consistent and stable model in terms of performance across different data subsets. The least stable model is the ET model, which showed the highest SD values in all metrics. This shows that the performance of the ET model is more sensitive (with higher variance) to the subset of data on which it is trained and produces more fluctuating results in different “folds”. The RF model has slightly higher SD values for stability than the ANN model, but significantly lower than ET. This shows that RF combines high performance with relatively good stability. The RF and ET models reached exceptionally high “Max” values in some “folds”. This shows that both models are capable of near-perfect classification on certain subsets of the dataset. The “Max” values of the ANN model are lower than the others. In contrast, the “Min” values are higher than those of the ET model. This suggests that ANN operates within a narrower performance range (as supported by the lower SD), never peaking as much as RF or ET, but also not performing as poorly (compared to ET). The potential reasons for the results in this table can be discussed as based on the fundamental working principles of the models: RF and ET can easily capture nonlinear relationships between features. The poor performance of ANN may be due to having an unoptimized architecture or insufficient hyperparameter tuning. RF and ET methods are very resistant to overfitting. ANN, on the other hand, may have difficulty generalizing across “folds” if it is not regularized well enough or if its architecture is too complex. The fact that RF outperforms ET implies that finding the optimal splitting thresholds for features in this dataset is more important than performing completely random splitting. The excessive randomness of ET may have led to information loss and poorer performance in some “folds” (as indicated by the high SD). The fact that ANN has the lowest SD in all metrics indicates the model has low variance. However, when combined with its lower performance, this may suggest the model has a high bias or is underfitting. Instead of learning the complex patterns, the ANN might have learned a simpler, more generalized decision boundary that is consistent across all folds. This would lower the average performance while increasing the cross-fold consistency. It is a strong possibility that the model’s hyperparameters were not optimized for this specific dataset.

**Figure 6:**
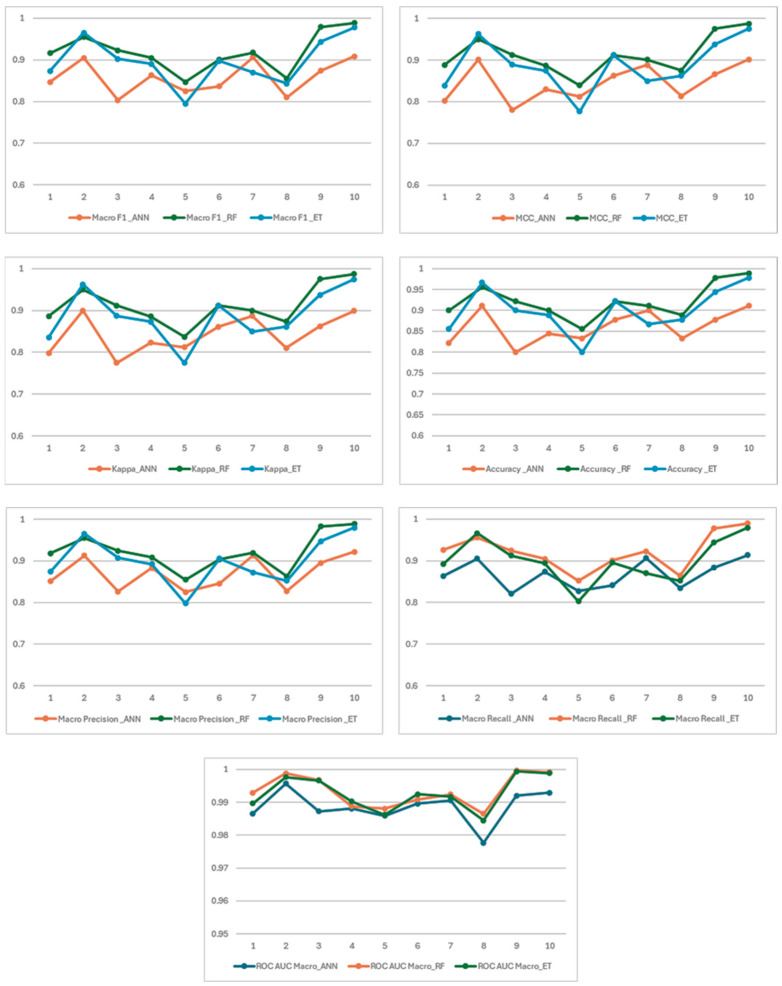
Fold basis metric results of ANN, ET, and RF models.

The fold-base metric results of each created ML model are shown in Figure 6. This comprehensive analysis on a balanced dataset demonstrates that the RF model significantly outperforms both ANN and ET across all evaluation metrics. RF achieves the highest average scores and the lowest variance, indicating its superior generalization ability and stability. The ET model also shows a robust performance, delivering near-perfect results in folds nine and ten (e.g., F1-score of 0.9778, accuracy of 97.78%, and Cohen’s Kappa of 0.9748 in fold ten). However, a notable performance drop is observed in fold five (F1: 0.7951, accuracy: 80.00%), suggesting that the ET model may be more sensitive to certain data subsets and exhibits slightly lower stability compared to the RF model. In contrast, the ANN model demonstrates relatively lower and more variable performance. The lower F1-scores in folds three and eight (0.8035 and 0.8102, respectively) and reduced accuracy in fold five (83.33%) are particularly noticeable. The average F1-score is approximately 0.847, the average accuracy is around 86.1%, and the average Cohen’s Kappa is about 0.830. These results suggest that the ANN model may not generalize as effectively on this dataset, or that more thorough hyperparameter tuning is required.

All experiments were performed on a personal workstation equipped with an Intel Core i7-3632QM CPU (2.2 GHz), 16 GB of RAM, and a Windows 10 operating system. No GPU acceleration was employed. The average total runtimes (including all ten folds) were as follows: Random Forest (RF) runtime was approximately 24 s, Extra Trees (ET) approximately 29 s, and Artificial Neural Network (ANN) approximately 55 s.

Even with ten-fold cross-validation, all models completed training within a short time, indicating that the proposed feature-based framework is computationally efficient and scalable. The experiments demonstrate that the models can be implemented effectively on standard hardware without requiring GPU acceleration or high-performance computing resources.

### 3.3. Based on Machine Learning Method and Evaluation Metrics

In this study, a multi-class dataset consisting of nine distinct genotypes of watermelon seeds was modeled using three ML algorithms: ANNs, RF, and ETs. A variety of statistical metrics were employed to comprehensively assess model performance, with k-fold cross-validation applied to mitigate overfitting and ensure robustness. Cross-validation provides more robust generalization capabilities by reducing the sensitivity of the obtained performance measurements to random differences in the training/test split [49]. The selected evaluation metrics capture not only classification accuracy but also class-wise consistency, model reliability, and discriminative capability, thus enabling a multi-faceted comparison across the three algorithms. The arithmetic mean of the metric results for the ANN, ET, and RF models is shown in Table 5. According to Table 5, when interpretations were made on the basis of machine learning methods and metrics, the following conclusions were reached.

The accuracy metric has been considered as a basic indicator because it reflects the success of the general classification. It shows the overall proportion of correctly classified instances [50]. While RF (0.9222) and ET (0.9000) achieved comparable and notably high accuracy scores, the ANN model lagged behind with a score of 0.8611. An accuracy value close to one indicates that the model correctly predicts classes with high success. According to the results, the classification success of RF and ET models is nearly 92% and 90%, respectively. Nonetheless, prior research has emphasized that accuracy alone can be misleading in multi-class scenarios, even when class balance exists [51]. Therefore, to gain deeper insights into model behavior, additional class-sensitive metrics were incorporated into the evaluation.

Macro-averaged precision, recall, and F1-score were selected due to their suitability for multi-class classification tasks. The fact that the values of these metrics are close to one shows us that the models are successful in the classification process; that is, if we explain specifically for the article, the classes of watermelon seeds are predicted correctly. It can be said that the models make errors in the classification process as they move away from the value one [33]. By averaging metric scores across classes without regard to their support, macro averaging ensures that each class contributes equally to the final measure—thus reducing the dominance of majority classes [52,53]. The F1-score reflects how well the model minimizes both false positive and false negative predictions by taking the harmonic mean between precision and recall [54]. Because the class-based recall and precision scores are high, the recall macro and precision macro have performed well. RF and ET demonstrated superior performance in terms of precision (0.9217 and 0.8994), recall (0.9220 and 0.9009), and F1-score (0.9187 and 0.8958). In contrast, ANN showed lower scores and a less balanced performance. These results underline the capacity of ensemble-based algorithms to learn complex decision boundaries more effectively than individual learners.

Beyond accuracy and class-level balance, this study also incorporates metrics that measure deviation from random chance. The Kappa coefficient quantifies agreement between predictions and ground truth, adjusted for chance agreement. If the model makes an excellent classification, the Kappa result of the model takes the value of one. RF (0.9118) and ET (0.8868) outperformed ANN (0.8428) in this regard.

Furthermore, Matthews Correlation Coefficient (MCC) was utilized for its ability to consider true and false positives and negatives comprehensively across all classes. MCC is often regarded as one of the most reliable evaluation metrics for both imbalanced and multi-class classification problems [55]. Again, RF (0.9127) and ET (0.8879) delivered stronger performances than ANN (0.8457). If the MCC value is near to one, the model’s predictions are accurate. The number of properly classified items has a significant influence on the MCC metric. As a result, any inaccuracy in one of the classes will significantly diminish the metric’s outcome. Classification mistakes made by the ANN model resulted in a lower MCC metric value than others.

The models’ ability to distinguish between classes was further evaluated using the ROC-AUC (Receiver Operating Characteristic—Area Under the Curve) metric, which was calculated in its macro-averaged form to treat all classes equally. In this metric, which takes values between zero and one, it can be interpreted as the higher the score, the better the model. Values close to one (especially 0.8 and above) indicate that the model has a high success rate in separating classes. RF achieved the highest AUC score (0.9934), followed closely by ET (0.9928) and ANN (0.9886). These results confirm the strong discriminative capability of all three models but also indicate a slight but consistent advantage of the tree-based ensemble methods over neural networks in terms of inter-class separability [56].

The results indicate that both the RF and ET models outperformed the ANN model across all evaluation metrics. This performance difference can be attributed primarily to the structure and data requirements of the models. While ANNs are highly flexible and powerful in capturing complex nonlinear relationships, they require large amounts of data and careful hyperparameter tuning to generalize effectively. In this study, despite the balanced dataset and cross-validation, the sample size within each of the nine seed classes may not have been sufficient for the ANN to achieve stable convergence. In contrast, ensemble tree-based methods such as RF and ET are inherently more robust to smaller datasets and can effectively handle nonlinearity and inter-feature dependencies without extensive parameter optimization.

Furthermore, the superior results of RF and ET can also be explained by their mechanisms for reducing overfitting and variance through randomization and averaging. Both models combine multiple decorrelated decision trees, improving generalizability and reducing sensitivity to noise—a crucial advantage when dealing with natural biological variability such as differences among watermelon seed classes. ANNs, on the other hand, can easily overfit when the feature dimensionality is moderate, but the sample size is limited, even with regularization techniques. Therefore, the higher stability and interpretability of tree-based ensembles make them better suited for the classification of morphological or spectral seed characteristics, where feature interactions are complex, but the dataset scale is constrained.

**Figure 7:**
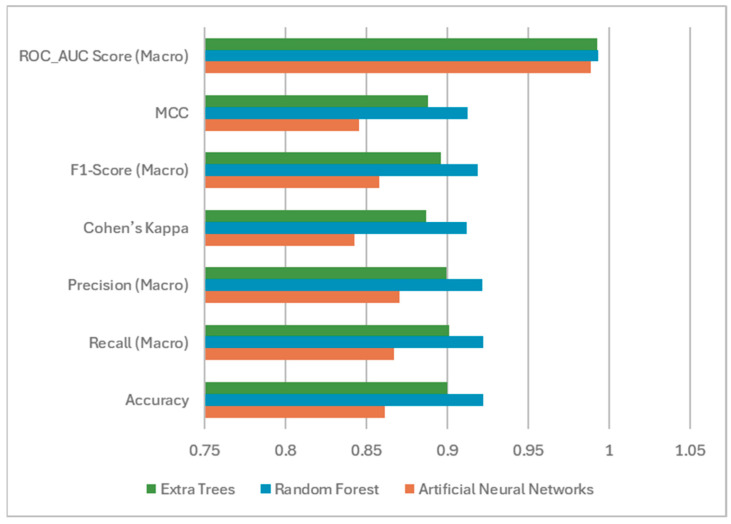
Bar graphs of the ANN, ET, and RF models.

Figure 7 displays the bar graph of the ML models. The bar chart shows that the RF model is more successful than the ANN and ET models.

### 3.4. Statistical Analysis: Significance Between Model Results

To evaluate whether the differences in performance between models were statistically significant for each model pair, a Wilcoxon Signed-Rank Test was applied to each metric and Benjamini–Hochberg correction was made. The test results are shown in Table 6. According to the test results, the following results have been achieved.

Accuracy: The Wilcoxon signed-rank test with Benjamini–Hochberg correction confirmed that both RF and ET significantly outperformed ANN (*p* = 0.015 and *p* = 0.015, respectively), indicating statistically significant differences. On the other hand, no significant difference (*p* = 0.161) was observed between RF and ET. These results demonstrate that ensemble tree-based models, particularly RF, provide superior overall classification performance in seed classification tasks on a balanced dataset.

Recall (Macro): Statistical analysis revealed that both RF and ET significantly outperformed ANN (RF vs. ANN: *p* = 0.015; ET vs. ANN: *p* = 0.015). Moreover, the difference between RF and ET is not statistically significant (*p* = 0.140). The metric value of RF is slightly higher, thus indicating that the RF model is more effective in minimizing false negatives and correctly detecting samples in all seed classes.

Precision (Macro): The comparison between models showed that both RF and ET significantly outperformed ANN (RF vs. ANN: *p* = 0.015; ET vs. ANN: *p* = 0.015). The difference between RF and ET is not statistically significant (*p* = 0.285). The metric result of RF is slightly higher; this highlights its superior ability to minimize false positives.

Cohen’s Kappa: Both RF and ET showed significantly higher Kappa scores than ANN (RF vs. ANN: *p* = 0.015; ET vs. ANN: *p* = 0.015). Moreover, the difference between RF and ET is not statistically significant (*p* = 0.161). The metric result of RF is slightly higher; this suggests that RF’s performance is not due to chance and reflects genuine predictive power.

Matthews Correlation Coefficient: Statistical tests confirmed that both RF and ET significantly outperformed ANN (RF vs. ANN: *p* = 0.015; ET vs. ANN: *p* = 0.015). Additionally, the difference between RF and ET is not significant (*p* = 0.161). The metric result of RF is slightly higher than others; this indicates that RF provides the most balanced and robust classification performance across all classes.

ROC-AUC Score (Macro): Both RF and ET significantly outperformed ANN (ET vs. ANN: *p* = 0.015; RF vs. ANN: *p* = 0.015), with the RF vs. ANN comparison approaching conventional significance thresholds. However, the difference between RF and ET is insignificant (*p* = 0.441). The metric result of RF is slightly higher than other ML models. This suggests that RF is exceptionally effective at ranking and separating seed classes probabilistically.

F1-Score (Macro): Both RF and ET significantly outperformed ANN. The difference in the F1-score between RF and ET is not statistically significant. Since the F1-score shows the balance of sensitivity and precision, it can be said that RF and ET provide this balance better. This result reinforces RF’s status as the most well-rounded and effective model for the seed classification task.

In our research work, the classification accuracy of 92.22% obtained by the Random Forest (RF) model proves to be highly competitive compared to other results that use deep learning (DL) techniques in the area of seed classification research in current years.

General research on ML and DL has proved that algorithms such as CNNs, SVM, and RF with features such as morphology, color, and texture can classify seeds with an accuracy ranging between 85 and 95%. Focusing on DL research specifically targeting watermelon seeds, there exists research that utilized X-ray imaging with the Resnet-50 transfer learning model that produced an accuracy of 87.3% [57]. Other research produced results with a classification accuracy of up to 87.97% that combined Ensemble DL models (LeNet, Resnet, and GoogLeNet). The results (92.22% accuracy with RF) indicate that traditional ML models such as RF are capable of greater success rates in non-destructive genotyping classification tasks involving seeds over more complex DL models. This is an indicator of the quality of the features that were carefully handpicked and the efficacy of the model to resist higher dimensionality and noise [16]. Although other research involving DL models targeting other agricultural items has proven to produce highly accurate results (97–99% accuracy in classing rice types and 100% accuracy in classing Maize types) [21], the balanced result produced by the classical ML model (RF) in this current multi-class problem targeting watermelon genotypes proves that simpler models are more advantageous in terms of efficiency.

Ahmed et al. [57] classified different types of watermelon seeds, comparing LDA and ResNet-50 transfer learning models, and reported the highest accuracy of 87.3% with ResNet-50. Gulzar et al. [58] achieved 99% accuracy, 0.99 recall, and 0.99 precision values when classifying different types of seeds. Ropelewska et al. [59] classified apricot stones using image analysis and ML, with some models reaching 100% accuracy, precision, recall, and F1-score. Qi et al. [16] classified watermelon seeds using a deep learning-based model, achieving an accuracy of 87.97% on the test set. Yurdakul et al. [60] used different types of CNN models for almond variety classification, achieving 0.99 accuracy, 0.99 precision, 0.99 recall, and 0.99 F1-score. Ercan et al. [33] used different types of ML techniques to classify Pitaya fruit. The highest accuracy (0.9866), precision macro (0.9823), recall macro (0.9891), F1-score macro (0.9870), Kappa (0.9809), and MCC (0.9817) were obtained by the RF model. A study conducted by Ermiş et al. [14], RF model yielded the best results. Accuracy, precision macro, recall macro, F1-score macro, MCC, and Kappa results were 0.9590, 0.9620, 0.9610, 0.9610, 0.9510, and 0.9510, respectively.

The dataset used in the study includes data obtained from 900 seeds (100 samples per class) belonging to nine classes. In this study, classical ML methods were preferred instead of CNN because CNNs often need large-scale datasets to achieve robust generalization. Some studies have shown that traditional ML methods often outperform deep learning methods when the dataset is relatively small. Deep learning architectures generally involve a large number of trainable parameters, which increases the risk of overfitting when training data are limited. In contrast, traditional models can provide stable performance and better generalization under such constraints [61,62].

In this study, while the RF and ET models produced similar outcomes across all metrics, the RF model performed marginally better. The ANN model was the least successful model. The best mean accuracy, recall (macro), precision (macro), F1-score (macro), MCC, Cohen’s Kappa, and ROC-AUC score results were 0.9222, 0.9220, 0.9217, 0.9187, 0.9127, 0.9118, 0.9934, respectively. The ML models’ results show that the snack-type watermelon seeds were successfully classified.

## 4. Conclusions

This study illustrates that the integration of morphological descriptors with advanced machine learning algorithms offers a reliable, non-destructive, and scalable methodology for the classification of snack-type watermelon (*Citrullus lanatus*) genotypes. By utilizing detailed seeds’ morphological and colorimetric features, the developed models achieved high classification accuracies, outperforming traditional optical sorting and manual inspection techniques. The findings underscore the potential of ML-based frameworks to enhance varietal traceability, facilitate selection within breeding programs, and improve quality control in commercial seed production. In addition to their practical applications, these results contribute to the broader academic discourse advocating for the incorporation of computer vision and artificial intelligence within horticultural seed science. Given the global emphasis on sustainable, plant-based nutrition and the cultural and nutritional significance of snack-type watermelon seeds, the adoption of such technologies may yield both economic and quality advantages throughout the seed supply chain. Future research endeavors could expand upon this approach by integrating hyperspectral imaging, 3D morphology, or larger genotype datasets to further enhance model robustness and transferability across diverse production environments.

Previous methods of seed classification, such as visual inspection, morphological measurements, and even optical sorting, are hardly enough to differentiate closely related watermelon genotypes. This is particularly true in cases where morphological variation is minor and overlapping. Even trained specialists attain only 70–80% accuracy. This has made breeding programs and commercial seed production inconsistent and imprecise. This study is novel in presenting a scalable, objective, and non-destructive Machine Learning (ML) framework to accurately distinguish nine different snack-type watermelon genotypes (92.22% accuracy). This study uses high-resolution imaging, advanced digital measurement techniques, and an integrated approach combining morphological, physical, and colorimetric attributes. This greatly augments the reliability of genotype classification as well as the efficiency in identifying sophisticated seed morphology attributes that are likely to go unnoticed by the human eye. Rather than focusing on what is practical, this study complies with watermelon seed phenotyping industrial standards in quality control by providing an innovative solution to seed classification.

The robustness of the classification performance achieved in this study is supported not only by its high success rate but also by its ability to generalize to previously unseen data. This demonstrates that the model captures underlying patterns in seed morphology and color characteristics, rather than the noise of the dataset. Comparisons with traditional methods indicate a framework that overcomes the potential inconsistencies arising from human fatigue and subjectivity, reducing the inconsistencies and error rates often seen in manual processes. These results demonstrate that this machine learning approach complements existing technologies by addressing the limitations that even the most advanced optical sorting systems may encounter for features that extend beyond the surface. The application of this approach extends beyond the laboratory environment, particularly addressing the requirements of high throughput and precision processing in fields such as seed breeding and quality control. Potential future expansions include the integration of hyperspectral and 3D imaging, deepening the model’s capabilities to include both chemical and structural properties of the seed by considering a more comprehensive dataset. This forward-looking perspective aims to create more robust and reliable systems by contributing to the development of technology in agricultural sciences.

According to the results and graphs of ML models, the classification is successful. While the difference between the ANN model’s and other ML models’ results is significant, the difference between the ET and RF models’ results is insignificant. Tuning parameter adjustment is very important in ML methods and has a significant effect on the results.

## Figures and Tables

**Table 1:** List of watermelon genotypes and their seed coat colors.

Color Group	Genotype Code	Genotype ID	Description
Red	GSR-49-22	K1	Red seed coat
	GSR-71-22	K2	Red seed coat
	GSR-113-22	K3	Red seed coat
White	GSW-87-22	B1	White seed coat
	GSW-17-22	B2	White seed coat
	GSW-47-22	B3	White seed coat
Black	GSB-1-22	B1	Black seed coat
	GSB-2-22	B2	Black seed coat
	GSB-11-22	B3	Black seed coat

**Table 2:** Parameter settings of the ANN, ET, and RF models.

Model	Parameter Settings
ANN	hidden_layer_sizes = (64,128, 64), activation = ‘relu’, solver = ‘sgd’, alpha = 0.0001, batch_size = ‘auto’, learning_rate = ‘adaptive’, max_iter = 3000, learning_rate_init = 0.001,
ET	n_estimators = 250, criterion = ‘log_loss’, max_depth = 10, min_samples_split = 2, min_samples_leaf = 1, min_weight_fraction_leaf = 0.0, max_features = ‘log2’, bootstrap = False,
RF	n_estimators = 250, criterion = ‘log_loss’, max_depth = 10, min_samples_split = 2, min_samples_leaf = 1, min_weight_fraction_leaf = 0.0, max_features = ‘log2’, min_impurity_decrease = 0.0, verbose = 0, ccp_alpha = 0.0, bootstrap = True,

**Table 3:** Descriptive statistics of the data.

	Average	SD	Min	Max	Skewness	Kurtosis
Length (mm)	13.62	1.04	10.32	16.88	0.65	0.77
Width (mm)	8.35	0.58	6.51	10.23	−0.12	−0.03
Thickness (mm)	2.42	0.23	1.87	3.70	0.56	1.14
Mass (g)	0.14	0.03	0.04	0.21	0.00	0.22
L	50.98	18.95	25.67	85.88	0.60	−1.33
a	10.28	7.68	1.46	29.10	1.01	−0.48
b	11.25	7.90	−2.90	27.71	−0.48	−1.32
Area (mm^2^)	89.60	11.31	53.33	121.31	0.30	0.24
Perimeter (mm)	35.03	2.31	26.87	41.79	0.33	0.58
Ratio of Height/Width	1.64	0.12	1.25	2.01	0.17	−0.02
Compactness	6.56	0.46	5.11	8.03	−0.12	−0.03
Roundness	0.91	0.02	0.84	0.98	−0.29	−0.07
Equivalent diameter (mm)	10.66	0.67	8.24	12.43	0.09	0.35

**Table 4:** Metric based statistics of fold results of ANN, RF, and ET models.

		ANN	RF	ET		ANN	RF	ET
Macro F1	Min	0.8035	0.8470	0.7951	Macro Precision	0.8251	0.8546	0.7981
	Max	0.9082	0.9889	0.9778		0.9216	0.9889	0.9798
	Mean	0.8578	0.9187	0.8958		0.8702	0.9217	0.8994
	SD	0.0398	0.0467	0.0557		0.0393	0.0446	0.0549
MCC	Min	0.7802	0.8392	0.7764	Macro Recall	0.8206	0.8524	0.8025
	Max	0.9018	0.9875	0.9753		0.9139	0.9899	0.9798
	Mean	0.8457	0.9127	0.8879		0.8670	0.9220	0.9009
	SD	0.0438	0.0461	0.0609		0.0349	0.0445	0.0534
Kappa	Min	0.7745	0.8372	0.7749	Accuracy	0.8000	0.8556	0.8000
	Max	0.8997	0.9874	0.9748		0.9111	0.9889	0.9778
	Mean	0.8428	0.9118	0.8868		0.8611	0.9222	0.9000
	SD	0.0449	0.0467	0.0614		0.0396	0.0412	0.0544

**Table 5:** Metrics results for ANN, ET, and RF models.

Metrics	Artificial Neural Networks	Random Forest	Extra Trees
Accuracy	0.8611	0.9222	0.9000
Recall (Macro)	0.8670	0.9220	0.9009
Precision (Macro)	0.8702	0.9217	0.8994
Cohen’s Kappa	0.8428	0.9118	0.8868
F1-Score (Macro)	0.8578	0.9187	0.8958
Matthews Correlation Coefficient	0.8457	0.9127	0.8879
ROC-AUC Score (Macro)	0.9886	0.9934	0.9928

**Table 6:** Table of significance between models.

Metrics	Comparison Models	Wilcoxon Signed-Rank Test with Benjamini–Hochberg Correction	Significant
Accuracy	RF vs. ANNs	0.015	+
	ET vs. ANNs	0.015	+
	RF vs. ET	0.161	-
Recall (Macro)	RF vs. ANNs	0.015	+
	ET vs. ANNs	0.015	+
	RF vs. ET	0.140	-
Precision (Macro)	RF vs. ANNs	0.015	+
	ET vs. ANNs	0.015	+
	RF vs. ET	0.285	-
Cohen’s Kappa	RF vs. ANNs	0.015	+
	ET vs. ANNs	0.015	+
	RF vs. ET	0.161	-
Matthews Correlation Coefficient	RF vs. ANNs	0.015	+
	ET vs. ANNs	0.015	+
	RF vs. ET	0.161	-
ROC-AUC Score (Macro)	RF vs. ANNs	0.015	+
	ET vs. ANNs	0.015	+
	RF vs. ET	0.441	-
F1-Score (Macro)	RF vs. ANNs	0.015	+
	ET vs. ANNs	0.015	+
	RF vs. ET	0.139	-

## Data Availability

The data presented in this study are available in the article and Supplementary Material. Additional raw data supporting the findings of this study are available from the corresponding author upon reasonable request.

## Supplementary Materials

The following supporting information can be downloaded at: https://www.mdpi.com/article/10.3390/foods14234069/s1. Supplementary File S1: Minimal dataset representing 30% of the raw data used in this study (including morphological and colorimetric seed parameters).

## Abbreviations

ANN	Artificial Neural Networks
RF	Random Forest
ET	Extra Trees
ML	Machine Learning
CNNs	Convolutional Neural Networks
SVM	Support Vector Machines
LR	Logistic Regression
kNN	k-Nearest Neighbors
NIR-HSI	Near-Infrared Hyperspectral Imaging
