# Supplementary material for: Identification and Classification of Snack-Type Watermelon (Citrullus lanatus) Genotypes Using Seed Morphology and Machine Learning Techniques
Source: Foods. 2025 Nov 27;14(23):4069. doi: 10.3390/foods14234069 (PMC12692002; doi:10.3390/foods14234069)
Supplement: Supplementary file 1 [file foods-3959650-supplementary.pdf › foods-3959650-supplementary.pdf]

Dear Editor,

We are sending 30% of the data we used in the article as a table below. The variable marked in yellow is the target variable and the ones in blue are the explanatory variable.

The raw data supporting the conclusions of this article will be made available by the authors on request.

Best regards.

Genotype	Length	Width	Thickness	Weight	L	a	b	Area	Perimeter	Ratio of Height/Width	Compactness	Roundness	Equivalent Diameter
GSW-087-22	14.36	8.06	2.6	0.13	79.67	4.36	13.97	90.90	35.92	1.78	6.33	0.89	10.76
GSB-001-22	14.05	8.04	2.35	0.16	37.23	4.68	5.67	88.72	35.34	1.75	6.31	0.89	10.63
GSB-001-22	13.79	7.91	2.22	0.14	30.93	5	2.9	85.67	34.71	1.74	6.21	0.89	10.44
GSR-113-22	13.05	8.75	2.28	0.13	41.83	19.37	14.1	89.68	34.58	1.49	6.87	0.94	10.69
GSB-002-22	12.97	7.53	2.15	0.13	39.13	2.83	-1.83	76.71	32.77	1.72	5.91	0.90	9.88
GSR-113-22	13.52	8.36	2.29	0.14	36.85	22.85	13.03	88.77	34.85	1.62	6.57	0.92	10.63
GSR-049-22	12.35	8.37	2.08	0.14	43.71	28.09	17.65	81.19	32.85	1.48	6.57	0.95	10.17
GSW-017-22	14.37	9.34	2.29	0.15	77.13	5.28	17.62	105.41	37.66	1.54	7.34	0.93	11.59
GSR-071-22	13.27	8.07	2.7	0.14	48.2	10.85	12.86	84.11	34.02	1.64	6.34	0.91	10.35
GSB-001-22	13.49	7.29	2.14	0.13	35.65	5.03	1.91	77.24	33.37	1.85	5.73	0.87	9.92
GSR-049-22	13.89	8.19	2.43	0.16	42.65	22.88	14.94	89.35	35.26	1.70	6.43	0.90	10.67
GSW-017-22	13.69	9.89	2.48	0.15	78.78	4.93	17.74	106.34	37.28	1.38	7.77	0.96	11.64
GSR-049-22	12.7	8.32	2.17	0.12	40.86	21.63	16.47	82.99	33.38	1.53	6.53	0.94	10.28
GSB-002-22	12.47	7.41	2.26	0.13	33.24	3.37	-1.42	72.57	31.74	1.68	5.82	0.91	9.61
GSB-001-22	11.39	7.04	2.08	0.10	43.02	2.78	0.5	62.98	29.35	1.62	5.53	0.92	8.95
GSB-001-22	13.76	7.89	2.34	0.15	31.77	4.15	1.86	85.27	34.64	1.74	6.20	0.89	10.42
GSR-113-22	13.34	8.98	2.08	0.16	36.8	20.01	10.95	94.09	35.40	1.49	7.05	0.94	10.95
GSB-002-22	12.46	7.5	2.06	0.12	45.93	2.56	-2.73	73.40	31.84	1.66	5.89	0.91	9.67
GSW-017-22	14.06	7.96	2.76	0.09	81.23	4.5	13.52	87.90	35.26	1.77	6.25	0.89	10.58
GSB-001-22	12.25	7.16	2.15	0.10	35.73	2.52	0.14	68.89	31.02	1.71	5.62	0.90	9.37
GSW-087-22	14.65	8.54	2.4	0.12	82.31	4.52	15.98	98.26	37.06	1.72	6.71	0.90	11.19
GSR-113-22	13.56	8.74	2.33	0.16	39.22	21.72	13.18	93.08	35.44	1.55	6.86	0.93	10.89
GSR-071-22	12.71	8.76	2.9	0.17	39.77	14.64	18.46	87.45	34.01	1.45	6.88	0.95	10.55
GSB-002-22	12.73	7.62	2.26	0.13	29.18	3.73	-1.39	76.19	32.47	1.67	5.98	0.91	9.85
GSW-047-22	15.08	9.38	2.67	0.20	73.11	8.33	19.42	111.10	38.95	1.61	7.37	0.92	11.89
GSR-049-22	13.7	7.91	2.62	0.15	39.95	24.91	15.53	85.11	34.56	1.73	6.21	0.90	10.41
GSR-071-22	13.17	8.38	2.81	0.14	45.86	11.97	19.31	86.68	34.27	1.57	6.58	0.93	10.51
GSW-087-22	13.93	8.83	2.5	0.08	82.28	4.33	13.31	96.61	36.20	1.58	6.94	0.93	11.09
GSW-017-22	14.49	9.41	2.25	0.15	78.17	4.89	16.09	107.09	37.97	1.54	7.39	0.93	11.68
GSB-001-22	12.01	7.59	2.27	0.13	38.8	3.97	0.06	71.59	31.18	1.58	5.96	0.93	9.55
GSR-113-22	12.91	8.82	2.08	0.15	43.03	24.37	16.2	89.43	34.44	1.46	6.93	0.95	10.67
GSB-002-22	11.95	7.88	2.21	0.13	31.8	4.99	-0.7	73.96	31.48	1.52	6.19	0.94	9.70
GSB-011-22	13.78	8.25	2.54	0.11	33.33	5.75	1.1	89.29	35.15	1.67	6.48	0.91	10.66
GSW-017-22	13.92	9.21	2.45	0.14	78.09	4.94	15.69	100.69	36.71	1.51	7.23	0.94	11.32
GSW-017-22	14.89	8.32	3.38	0.16	78.55	5.52	18.55	97.30	37.19	1.79	6.53	0.88	11.13
GSR-049-22	13.77	8.35	2.33	0.16	46.43	18.73	9.01	90.30	35.27	1.65	6.56	0.91	10.72
GSR-113-22	13.23	8.28	2.67	0.16	46.41	25.85	16.65	86.04	34.24	1.60	6.50	0.92	10.47

GSW-047-22	15.34	9.05	2.33	0.19	69.75	8.91	22.32	109.03	38.95	1.70	7.11	0.90	11.78
GSW-017-22	12.17	7.46	2.22	0.06	80.94	5.02	13.07	71.31	31.28	1.63	5.86	0.92	9.53
GSR-049-22	13.48	7.67	2.54	0.15	42.98	25.81	21.93	81.20	33.85	1.76	6.02	0.89	10.17
GSB-011-22	13.25	7.88	2.1	0.09	34.14	8.93	2.7	82.00	33.73	1.68	6.19	0.91	10.22
GSR-049-22	13.15	7.96	2.83	0.15	41.28	17.6	9.01	82.21	33.66	1.65	6.25	0.91	10.23
GSB-001-22	13.53	7.73	2.31	0.14	36.4	4.16	3.29	82.14	34.02	1.75	6.07	0.89	10.23
GSW-017-22	14.35	7.44	2.3	0.15	80.92	4.68	15.61	83.85	35.09	1.93	5.84	0.86	10.33
GSB-011-22	13.19	8.21	2.44	0.10	32.79	6.96	0.6	85.05	34.07	1.61	6.45	0.92	10.41
GSW-047-22	15.45	9.22	2.62	0.20	72.23	7.94	19.97	111.88	39.37	1.68	7.24	0.91	11.94
GSB-011-22	12.77	8.45	2.31	0.10	33.41	7.31	1.45	84.75	33.68	1.51	6.64	0.94	10.39
GSR-113-22	13.55	8.86	2.22	0.16	43.66	24.3	18.72	94.29	35.59	1.53	6.96	0.94	10.96
GSB-011-22	13.7	8.67	2.23	0.13	30.68	6.03	1.26	93.29	35.58	1.58	6.81	0.93	10.90
GSW-047-22	15.98	8.46	2.8	0.20	68.74	7.69	17.4	106.18	39.30	1.89	6.64	0.86	11.63
GSR-071-22	12.81	8.5	2.63	0.15	48.44	9.87	11.09	85.52	33.82	1.51	6.68	0.94	10.43
GSW-017-22	13.53	9.46	2.47	0.11	80.37	4.94	14.07	100.53	36.40	1.43	7.43	0.95	11.31
GSR-113-22	13.64	8.36	2.54	0.16	43.91	23.1	19.82	89.56	35.06	1.63	6.57	0.92	10.68
GSR-049-22	13.29	7.82	2.51	0.15	40.77	26.1	16.26	81.62	33.72	1.70	6.14	0.90	10.19
GSR-113-22	13.26	8.59	2.26	0.16	44.02	23.15	20.11	89.46	34.71	1.54	6.75	0.93	10.67
GSR-113-22	13.82	7.97	2.54	0.16	49.08	18.63	9.32	86.51	34.85	1.73	6.26	0.90	10.50
GSW-087-22	14.48	8.23	2.74	0.10	77.54	4.36	11.32	93.60	36.35	1.76	6.46	0.89	10.92
GSW-087-22	14.72	9.14	2.42	0.11	81.27	4.86	14.85	105.67	37.99	1.61	7.18	0.92	11.60
GSB-001-22	12.72	7.13	2.17	0.12	35.72	4.71	4.17	71.23	31.80	1.78	5.60	0.89	9.52
GSW-017-22	14.66	9.38	2.35	0.14	76.96	5.44	18.55	108.00	38.22	1.56	7.37	0.93	11.73
GSW-017-22	13.7	9.68	2.76	0.16	78.58	5.34	17.94	104.16	37.00	1.42	7.60	0.96	11.52
GSR-049-22	13.51	7.61	2.52	0.15	39.53	25.5	14.24	80.75	33.83	1.78	5.98	0.89	10.14
GSR-049-22	13.54	8.27	2.63	0.15	39.34	26.56	21.45	87.95	34.76	1.64	6.50	0.91	10.58
GSW-087-22	14.21	8.38	2.43	0.04	84.18	3.77	17.76	93.53	36.08	1.70	6.58	0.90	10.91
GSW-087-22	13.7	8.32	2.42	0.14	79.7	4.67	13.8	89.52	35.11	1.65	6.53	0.91	10.68
GSW-017-22	13.13	8.88	2.52	0.08	83.65	4.02	16.14	91.57	34.90	1.48	6.97	0.94	10.80
GSB-001-22	13.64	7.34	2.29	0.14	32.91	4.75	2.21	78.63	33.70	1.86	5.76	0.87	10.01
GSW-087-22	12.24	7.39	2.92	0.10	80.05	4.17	12.34	71.04	31.31	1.66	5.80	0.91	9.51
GSW-047-22	15.55	9.14	2.57	0.19	72.25	8.43	20.31	111.63	39.44	1.70	7.18	0.90	11.92
GSR-071-22	13.02	8.64	2.55	0.16	41.1	14.47	13.47	88.35	34.37	1.51	6.79	0.94	10.61
GSB-002-22	12.74	7.55	2.13	0.13	35.82	3.37	-1.46	75.55	32.39	1.69	5.93	0.90	9.81
GSR-113-22	13.18	8.4	2.52	0.15	40.16	24.04	14.46	86.95	34.31	1.57	6.60	0.93	10.52
GSB-001-22	11.5	6.51	2.31	0.10	35.63	2.56	0.93	58.80	28.84	1.77	5.11	0.89	8.65
GSB-002-22	12.18	8.37	2.43	0.13	28.72	3.63	-0.88	80.07	32.56	1.46	6.57	0.95	10.10
GSR-113-22	13.33	8.71	1.96	0.12	43.08	18.51	14.23	91.19	35.00	1.53	6.84	0.94	10.78
GSR-049-22	13.7	7.59	3.05	0.15	37.71	25.93	16.18	81.67	34.13	1.81	5.96	0.88	10.20

GSB-001-22	13.11	8.13	2.07	0.14	30.77	4.94	2.82	83.71	33.82	1.61	6.39	0.92	10.32
GSB-002-22	11.76	7.79	2.1	0.12	29.1	4.62	-0.41	71.95	31.03	1.51	6.12	0.94	9.57
GSB-001-22	12.92	8.3	2.16	0.14	34.53	4.58	3.85	84.22	33.73	1.56	6.52	0.93	10.36
GSW-017-22	14.47	8.41	2.35	0.15	79.11	5.8	17.21	95.58	36.57	1.72	6.61	0.90	11.03
GSR-071-22	11.87	7.77	2.2	0.13	43.65	13.84	17.44	72.44	31.19	1.53	6.10	0.94	9.60
GSW-017-22	12.78	9.38	2.6	0.14	81.74	5.18	14	94.15	35.01	1.36	7.37	0.97	10.95
GSB-002-22	13.41	7.69	2.3	0.14	34.1	3.37	-1.56	80.99	33.76	1.74	6.04	0.89	10.15
GSB-011-22	13.61	8.61	2.28	0.13	31.64	6.64	1.39	92.03	35.35	1.58	6.76	0.93	10.83
GSB-001-22	14.16	7.74	2.22	0.15	42.93	3.94	0.93	86.08	35.14	1.83	6.08	0.88	10.47
GSB-002-22	13.64	7.74	2.26	0.14	29.07	3.59	-1.27	82.92	34.23	1.76	6.08	0.89	10.27
GSW-017-22	14.59	9.21	2.44	0.16	79.62	5.6	19.88	105.54	37.86	1.58	7.23	0.93	11.59
GSB-011-22	14.01	8.17	2.23	0.11	34.13	5.77	1.11	89.90	35.45	1.71	6.42	0.90	10.70
GSR-113-22	13.11	8.85	2.06	0.15	44.2	21.74	16.03	91.12	34.82	1.48	6.95	0.94	10.77
GSR-113-22	12.8	8.86	2.2	0.15	45.52	22.51	12.91	89.07	34.31	1.44	6.96	0.95	10.65
GSR-071-22	12.63	8.77	2.27	0.15	43.17	12.05	27.71	86.99	33.89	1.44	6.89	0.95	10.52
GSR-049-22	13.98	8.24	2.39	0.16	38.92	22.68	14.66	90.47	35.49	1.70	6.47	0.90	10.73
GSW-087-22	14.17	8.56	2.44	0.14	78.24	5.03	17.73	95.27	36.25	1.66	6.72	0.91	11.01
GSR-113-22	13.66	8.72	2.41	0.16	39.29	20.59	11.24	93.55	35.58	1.57	6.85	0.93	10.91
GSB-001-22	13.36	7.88	2.18	0.14	30.95	5.38	3.41	82.68	33.92	1.70	6.19	0.90	10.26
GSR-113-22	13.91	8.63	2.4	0.16	44.05	23.89	18.38	94.28	35.89	1.61	6.78	0.92	10.96
GSR-049-22	13.22	7.78	2.51	0.15	43.43	25.14	20.51	80.78	33.54	1.70	6.11	0.90	10.14
GSW-047-22	15.69	9.49	2.61	0.20	71.92	8.5	20.32	116.94	40.15	1.65	7.45	0.91	12.20
GSR-113-22	14.32	8.77	2.4	0.17	43.97	24	17.94	98.64	36.80	1.63	6.89	0.92	11.21
GSW-017-22	15.69	8.64	2.67	0.16	78.63	5.5	18.6	106.47	39.02	1.82	6.79	0.88	11.64
GSB-001-22	12.85	7.9	2.27	0.14	41.14	4.21	7.22	79.73	33.06	1.63	6.20	0.92	10.08
GSR-071-22	13.4	8.3	2.54	0.16	44.56	13.13	12.34	87.35	34.56	1.61	6.52	0.92	10.55
GSR-049-22	13.18	8.23	2.56	0.15	42.5	26.39	19.72	85.19	34.08	1.60	6.46	0.92	10.41
GSW-047-22	16.19	9.34	2.72	0.21	69.58	8.25	22.24	118.76	40.83	1.73	7.34	0.90	12.30
GSR-071-22	13.09	7.5	2.97	0.16	42.96	13.01	11.83	77.11	32.94	1.75	5.89	0.89	9.91
GSB-001-22	13.36	7.64	2.51	0.14	32.01	4.4	3.1	80.17	33.60	1.75	6.00	0.89	10.10
GSR-071-22	12.78	8.36	2.53	0.17	44.4	11.01	16.69	83.91	33.57	1.53	6.57	0.94	10.34
GSW-087-22	13.56	8.8	2.48	0.08	84.22	4.71	14.06	93.72	35.52	1.54	6.91	0.93	10.92
GSB-002-22	12.48	7.85	2.21	0.13	28.39	3.98	-0.98	76.94	32.35	1.59	6.17	0.92	9.90
GSR-071-22	13.1	8.37	2.51	0.15	40.22	14.96	16.64	86.12	34.14	1.57	6.57	0.93	10.47
GSR-071-22	13.45	8.5	2.5	0.17	45.05	12.67	15.48	89.79	34.92	1.58	6.68	0.93	10.69
GSR-071-22	13.5	8.16	2.65	0.15	40.59	14.87	16.79	86.52	34.54	1.65	6.41	0.91	10.50
GSB-011-22	13.17	8.49	2.2	0.11	31.35	6.18	1.41	87.82	34.42	1.55	6.67	0.93	10.57
GSR-071-22	12.5	8.65	2.43	0.16	40.86	13.8	12.58	84.92	33.50	1.45	6.79	0.95	10.40
GSW-047-22	15.35	8.95	2.5	0.18	71.74	8.33	21.49	107.90	38.84	1.72	7.03	0.90	11.72

GSW-047-22	14.44	8.99	2.67	0.19	72.69	8.4	21.09	101.96	37.30	1.61	7.06	0.92	11.39
GSW-017-22	14.58	8.81	3.08	0.14	79.11	5.11	18.56	100.88	37.30	1.65	6.92	0.91	11.33
GSR-113-22	14.2	8.59	2.44	0.17	45.47	23.81	22.7	95.80	36.34	1.65	6.75	0.91	11.04
GSB-002-22	13.07	8.31	2.34	0.15	27.53	4.1	-0.36	85.30	34.00	1.57	6.53	0.93	10.42
GSR-049-22	13.78	7.89	2.45	0.16	41.63	24.73	18.64	85.39	34.67	1.75	6.20	0.89	10.43
GSB-001-22	13.8	8.1	2.16	0.14	41.4	4.46	2.5	87.79	34.99	1.70	6.36	0.90	10.57
GSR-049-22	12.85	8.5	2.54	0.15	41.26	25.68	20.21	85.79	33.89	1.51	6.68	0.94	10.45
GSR-049-22	12.98	8.08	2.53	0.14	45.55	25.66	17.65	82.37	33.53	1.61	6.35	0.92	10.24
GSR-049-22	12.49	7.93	2.38	0.13	39.28	25.36	17.78	77.79	32.48	1.58	6.23	0.93	9.95
GSB-002-22	12.77	7.49	2.16	0.13	31.85	3.49	-1.56	75.12	32.37	1.70	5.88	0.90	9.78
GSB-001-22	13.32	7.79	2.46	0.15	30.94	4.41	2.45	81.50	33.73	1.71	6.12	0.90	10.19
GSW-047-22	15.62	9.25	2.5	0.21	71.81	8.38	21.14	113.48	39.71	1.69	7.26	0.90	12.02
GSW-047-22	14.72	8.43	2.36	0.17	72.91	8.08	20.88	97.46	37.04	1.75	6.62	0.89	11.14
GSR-071-22	12.34	8.01	2.32	0.14	40.96	13.7	15.6	77.63	32.33	1.54	6.29	0.93	9.94
GSW-087-22	14.58	8.54	2.22	0.11	82.75	4.76	12.54	97.79	36.94	1.71	6.71	0.90	11.16
GSR-113-22	13.55	8.34	2.6	0.16	43.07	24.57	16.25	88.76	34.87	1.62	6.55	0.92	10.63
GSW-017-22	15.37	8.5	2.37	0.15	78.29	5.17	17.4	102.61	38.28	1.81	6.68	0.88	11.43
GSB-001-22	13.6	7.68	2.28	0.14	32.13	4.91	3.39	82.03	34.08	1.77	6.03	0.89	10.22
GSR-071-22	13.02	8.57	2.52	0.15	46.12	10.81	17.69	87.64	34.27	1.52	6.73	0.94	10.56
GSR-113-22	13.51	8.39	2.97	0.15	38.62	23.43	15.62	89.02	34.87	1.61	6.59	0.92	10.65
GSR-113-22	13.39	8.44	2.25	0.15	41.38	23	13.69	88.76	34.73	1.59	6.63	0.92	10.63
GSW-017-22	14.19	9.13	2.52	0.15	77.4	5.4	20.25	101.75	37.06	1.55	7.17	0.93	11.38
GSB-011-22	13.49	8.9	2.39	0.13	34.6	6.53	2.36	94.30	35.54	1.52	6.99	0.94	10.96
GSW-087-22	14.18	8.11	2.32	0.11	83.57	4.53	12.48	90.32	35.67	1.75	6.37	0.89	10.72
GSW-087-22	14.31	8.58	2.82	0.13	78.4	4.6	17.4	96.43	36.52	1.67	6.74	0.91	11.08
GSW-087-22	12.67	8.16	2.58	0.08	79.04	5.21	14.52	81.20	33.10	1.55	6.41	0.93	10.17
GSB-011-22	13.53	8.5	2.29	0.12	29.95	6.27	1.56	90.32	35.06	1.59	6.68	0.92	10.72
GSR-071-22	12.94	8.02	2.5	0.15	40.09	14.67	14.99	81.51	33.38	1.61	6.30	0.92	10.19
GSR-113-22	13.98	8.55	2.44	0.17	43.47	24.48	20.42	93.88	35.91	1.64	6.72	0.92	10.93
GSB-001-22	13.52	7.71	2.04	0.13	39.05	4.59	1.14	81.87	33.98	1.75	6.06	0.89	10.21
GSR-049-22	12.99	7.95	2.8	0.13	45.8	27.12	17.82	81.11	33.37	1.63	6.24	0.92	10.16
GSB-002-22	12.68	7.92	2.18	0.13	27.56	3.69	-1.39	78.87	32.79	1.60	6.22	0.92	10.02
GSR-113-22	13.36	8.49	2.19	0.13	40.23	20.54	13.23	89.09	34.75	1.57	6.67	0.93	10.65
GSR-049-22	12.97	8.32	2.19	0.14	40.69	27.21	16.84	84.75	33.84	1.56	6.53	0.93	10.39
GSB-011-22	12.37	8.5	2.8	0.11	29	6.83	1.96	82.58	33.06	1.46	6.68	0.95	10.25
GSW-017-22	15.16	8.58	2.46	0.16	75.16	6.13	20.38	102.16	38.01	1.77	6.74	0.89	11.40
GSW-047-22	16.19	9.26	2.4	0.20	70.56	8.7	21.96	117.75	40.72	1.75	7.27	0.89	12.24
GSR-049-22	12.6	8.5	2.16	0.14	42.23	26.38	20.47	84.12	33.46	1.48	6.68	0.94	10.35
GSW-017-22	15.3	8.6	2.47	0.16	75.93	6.13	18.28	103.34	38.28	1.78	6.75	0.89	11.47

GSR-049-22	13.31	7.89	2.6	0.16	41.3	25.15	17.39	82.48	33.85	1.69	6.20	0.90	10.25
GSB-002-22	12.56	7.53	2.18	0.13	32.68	2.55	-1.77	74.28	32.05	1.67	5.91	0.91	9.73
GSW-087-22	14.49	9.07	2.44	0.14	75.58	5.31	16.61	103.22	37.50	1.60	7.12	0.92	11.46
GSB-002-22	12.19	7.63	2.2	0.12	42.88	2.13	-2.64	73.05	31.55	1.60	5.99	0.92	9.64
GSW-047-22	15.38	9.44	2.58	0.20	72.64	7.99	19.93	114.03	39.55	1.63	7.41	0.92	12.05
GSW-047-22	15.23	8.63	2.27	0.17	73.61	8.1	17.8	103.23	38.20	1.76	6.78	0.89	11.46
GSW-087-22	13.89	8.5	2.46	0.12	80.02	4.31	12.49	92.73	35.68	1.63	6.68	0.92	10.87
GSB-001-22	14.02	7.82	2.22	0.14	34.98	5.43	4.23	86.11	35.00	1.79	6.14	0.88	10.47
GSW-017-22	15.38	8.79	2.65	0.16	72.78	5.83	19.18	106.18	38.68	1.75	6.90	0.89	11.63
GSR-071-22	13.1	8.6	2.49	0.16	46.77	14.35	18.54	88.48	34.45	1.52	6.75	0.94	10.61
GSB-001-22	13.4	7.7	2.34	0.14	29.38	4.1	0.76	81.04	33.75	1.74	6.05	0.89	10.16
GSB-011-22	12.53	8.29	2.31	0.11	32.08	6.61	0.78	81.58	33.04	1.51	6.51	0.94	10.19
GSB-002-22	12.57	7.78	2.12	0.13	34.98	3.65	-2.17	76.81	32.41	1.62	6.11	0.92	9.89
GSB-002-22	11.73	7.03	1.94	0.09	40.08	3.88	-2.22	64.77	29.93	1.67	5.52	0.91	9.08
GSB-002-22	11.4	7.13	2.14	0.10	42.65	2.3	-1.97	63.84	29.49	1.60	5.60	0.92	9.02
GSB-011-22	13.17	8.04	2.3	0.12	28.97	6.64	1.98	83.16	33.81	1.64	6.31	0.91	10.29
GSW-047-22	16.6	9.13	2.65	0.21	71.02	8.21	19.35	119.03	41.27	1.82	7.17	0.88	12.31
GSB-002-22	12.51	7.34	2.09	0.12	35.78	2.97	-1.77	72.12	31.71	1.70	5.76	0.90	9.58
GSB-001-22	14.12	7.39	2.78	0.15	33.42	5.34	3.13	81.95	34.62	1.91	5.80	0.86	10.22
GSR-071-22	12.76	8.73	2.84	0.17	42.72	14.88	19.3	87.49	34.05	1.46	6.86	0.95	10.55
GSB-002-22	12.35	7.44	2.14	0.11	29.94	4.23	-1.23	72.17	31.57	1.66	5.84	0.91	9.59
GSB-011-22	13.24	8.44	2.2	0.10	29.41	7.57	1.94	87.76	34.47	1.57	6.63	0.93	10.57
GSW-087-22	13.59	8.21	2.42	0.12	80	4.42	16.47	87.63	34.77	1.66	6.45	0.91	10.56
GSW-017-22	14.65	8.72	2.39	0.16	79.66	5.58	16.96	100.33	37.30	1.68	6.85	0.91	11.30
GSB-011-22	13.2	8.49	2.5	0.10	43	4.75	1.46	88.02	34.47	1.55	6.67	0.93	10.59
GSW-087-22	14.21	7.89	2.27	0.10	82.52	4.59	13.82	88.06	35.43	1.80	6.20	0.88	10.59
GSW-017-22	13.49	8.37	2.69	0.15	80.7	5.27	15.09	88.68	34.81	1.61	6.57	0.92	10.63
GSB-011-22	12.93	8.71	2.19	0.10	33.67	5.91	0.86	88.45	34.32	1.48	6.84	0.94	10.61
GSB-001-22	11.47	7.56	2.16	0.11	35.37	3.06	1.13	68.10	30.21	1.52	5.94	0.94	9.31
GSB-011-22	13.1	8.72	2.06	0.10	33.7	8.39	1.66	89.72	34.62	1.50	6.85	0.94	10.69
GSR-071-22	13.4	8.5	2.6	0.15	44.41	14.96	18.54	89.46	34.83	1.58	6.68	0.93	10.67
GSW-087-22	15.01	8.49	2.44	0.12	83.47	4.66	12.97	100.09	37.63	1.77	6.67	0.89	11.29
GSR-071-22	12.88	8.2	3.13	0.16	41.01	15.28	15.09	82.95	33.52	1.57	6.44	0.93	10.28
GSB-001-22	13.5	8.18	2.23	0.15	36.24	5.32	4.66	86.73	34.57	1.65	6.42	0.91	10.51
GSB-011-22	13.63	8.94	2.47	0.12	31.94	5.96	0.72	95.70	35.84	1.52	7.02	0.94	11.04
GSB-001-22	14.34	7.78	2.31	0.15	37.78	4.32	1.35	87.62	35.51	1.84	6.11	0.87	10.56
GSB-002-22	12.22	7.85	2.16	0.13	34.62	3.54	-1.98	75.34	31.90	1.56	6.17	0.93	9.79
GSW-047-22	15.5	8.42	2.72	0.19	69.96	8.49	20.36	102.50	38.40	1.84	6.61	0.87	11.42
GSB-011-22	12.7	8.69	2.02	0.08	33.09	10.67	3.95	86.68	33.90	1.46	6.83	0.95	10.51

GSB-002-22	12.91	7.25	2.27	0.13	32.43	2.3	-2.08	73.51	32.29	1.78	5.69	0.89	9.67
GSR-071-22	13.2	8.22	2.55	0.16	39.45	14.46	15.57	85.22	34.10	1.61	6.46	0.92	10.42
GSR-113-22	13.04	8.91	2.22	0.14	42.43	21.82	14.29	91.25	34.78	1.46	7.00	0.95	10.78
GSB-011-22	13.12	8.32	2.09	0.09	33.06	8.18	2.22	85.73	34.10	1.58	6.53	0.93	10.45
GSW-017-22	15.1	8.77	2.62	0.16	77.8	5.77	17.48	104.01	38.16	1.72	6.89	0.90	11.51
GSB-002-22	11.21	6.85	1.94	0.07	36.14	3.25	-1.39	60.31	28.78	1.64	5.38	0.91	8.76
GSR-113-22	14.02	7.97	2.53	0.17	45.21	23.46	15.69	87.76	35.20	1.76	6.26	0.89	10.57
GSW-047-22	14.41	8.65	2.76	0.18	72.99	8.12	19.63	97.90	36.79	1.67	6.79	0.91	11.16
GSB-001-22	13.73	7.97	2.38	0.15	41.26	4.28	1.45	85.94	34.69	1.72	6.26	0.90	10.46
GSW-017-22	13.9	9.22	2.45	0.15	76.02	4.64	17.32	100.66	36.69	1.51	7.24	0.94	11.32
GSW-017-22	14.56	9.41	2.33	0.16	77.96	5.39	17.5	107.61	38.09	1.55	7.39	0.93	11.71
GSR-113-22	13.57	8.34	2.49	0.15	39.49	26.33	16.54	88.89	34.91	1.63	6.55	0.92	10.64
GSR-113-22	13.63	8.42	2.46	0.16	41.73	24.8	20.09	90.14	35.12	1.62	6.61	0.92	10.71
GSR-113-22	13.84	8.44	2.46	0.16	45.24	22.93	16.58	91.74	35.51	1.64	6.63	0.91	10.81
GSW-087-22	13.7	8.22	2.8	0.08	80.96	4.69	13.74	88.45	34.97	1.67	6.46	0.91	10.61
GSW-087-22	14.23	8.22	2.43	0.12	79.54	5.12	17.95	91.87	35.90	1.73	6.46	0.90	10.82
GSB-001-22	13.7	7.73	2.19	0.14	32.47	3.95	2.97	83.17	34.32	1.77	6.07	0.89	10.29
GSW-017-22	13.74	9.7	2.45	0.16	79.94	5.04	16.2	104.68	37.09	1.42	7.62	0.96	11.54
GSR-113-22	13.71	8.47	2.25	0.14	37.16	20.9	13.01	91.20	35.33	1.62	6.65	0.92	10.78
GSW-047-22	15.18	8.5	2.65	0.17	73.85	8.11	19.33	101.34	37.94	1.79	6.68	0.88	11.36
GSR-071-22	12.9	8.26	2.58	0.15	40.26	14.18	15.66	83.69	33.64	1.56	6.49	0.93	10.32
GSR-113-22	13.46	8.65	2.36	0.13	43.25	16.19	10.33	91.44	35.14	1.56	6.79	0.93	10.79
GSW-017-22	14.38	8.88	2.43	0.15	72.9	6.38	19.46	100.29	37.05	1.62	6.97	0.92	11.30
GSW-017-22	14.73	8.48	3.08	0.16	79.43	4.75	15.78	98.10	37.12	1.74	6.66	0.89	11.18
GSB-002-22	12.4	7.69	2.31	13.02	26.62	3.3	-0.68	74.89	31.99	1.61	6.04	0.92	9.77
GSB-002-22	11.52	7.91	2.22	0.12	36.04	2.98	-2.18	71.57	30.78	1.46	6.21	0.95	9.55
GSR-049-22	13.4	8.24	2.09	0.14	43.41	25.23	20.08	86.72	34.48	1.63	6.47	0.92	10.51
GSW-047-22	15.5	9.28	2.58	0.20	71.44	8.35	20.83	112.97	39.54	1.67	7.29	0.91	11.99
GSW-047-22	16	8.52	2.56	0.19	70.4	8.44	21.1	107.07	39.42	1.88	6.69	0.87	11.68
GSB-001-22	13.02	7.71	2.24	0.14	33.12	4.46	2.72	78.84	33.10	1.69	6.06	0.90	10.02
GSW-017-22	13.95	8.2	2.57	0.14	79.8	4.46	14.33	89.84	35.38	1.70	6.44	0.90	10.70
GSR-049-22	13.8	7.9	2.58	0.15	39.66	22.98	11.31	85.62	34.72	1.75	6.20	0.89	10.44
GSR-071-22	12.25	8.52	2.8	0.16	41.41	10.18	23.22	81.97	32.89	1.44	6.69	0.95	10.22
GSR-071-22	13.21	8.23	2.61	0.16	40.38	13.98	18.29	85.39	34.13	1.61	6.46	0.92	10.43
GSW-017-22	14.68	8.03	3.19	0.15	75.84	5.2	18.5	92.58	36.44	1.83	6.31	0.88	10.86
GSB-002-22	12.16	7.28	2.16	0.11	29.84	3.87	-1.51	69.53	31.02	1.67	5.72	0.91	9.41
GSB-011-22	12.4	8.3	2.6	0.10	32.28	7.26	1.56	80.83	32.84	1.49	6.52	0.94	10.14
GSR-113-22	13.19	8.95	2.21	0.16	44.43	23.17	18.47	92.72	35.10	1.47	7.03	0.95	10.87
GSW-047-22	15.66	8.6	2.99	0.20	70.64	8.19	20.15	105.77	38.92	1.82	6.75	0.88	11.60

GSB-011-22	13.52	8.47	2.23	0.11	34.32	5.26	0.84	89.94	35.00	1.60	6.65	0.92	10.70
GSR-049-22	12.63	8.26	2.4	0.19	40.06	27.18	18.04	81.94	33.17	1.53	6.49	0.94	10.21
GSW-087-22	13.66	8.62	2.32	0.09	74.39	4.49	11.59	92.48	35.45	1.58	6.77	0.92	10.85
GSR-071-22	13	8.26	2.47	0.17	45.66	13.71	13.66	84.34	33.81	1.57	6.49	0.93	10.36
GSB-001-22	13.49	8.09	2.18	0.15	31.64	5.35	3.49	85.71	34.43	1.67	6.35	0.91	10.45
GSB-011-22	13.57	8.05	2.79	0.10	34.91	6.24	1.36	85.80	34.52	1.69	6.32	0.90	10.45
GSR-113-22	13.38	8.45	2.55	0.16	43.96	24.22	16.85	88.80	34.73	1.58	6.64	0.93	10.63
GSB-002-22	12.2	7.64	2.25	0.12	32.32	3.91	-1.43	73.21	31.58	1.60	6.00	0.92	9.65
GSR-113-22	13.33	8.39	2.24	0.16	41.42	27.92	19.04	87.84	34.56	1.59	6.59	0.92	10.58
GSW-017-22	15.3	8.33	2.46	0.16	77.44	5.71	16.6	100.10	37.93	1.84	6.54	0.87	11.29
GSW-087-22	13.02	7.78	2.44	0.09	83.19	3.95	11.54	79.56	33.19	1.67	6.11	0.91	10.06
GSR-071-22	13.94	8.66	2.45	0.17	44.73	13.38	18.97	94.81	35.99	1.61	6.80	0.92	10.99
GSW-087-22	14.06	8.5	2.48	0.09	82.53	4.24	17.65	93.86	35.98	1.65	6.68	0.91	10.93
GSW-017-22	14.13	9.54	2.41	0.15	79.57	4.86	17.37	105.87	37.53	1.48	7.49	0.94	11.61
GSB-011-22	13.27	8.65	2.05	0.11	30.28	6.22	1	90.15	34.82	1.53	6.79	0.93	10.71
GSR-049-22	13.77	7.68	2.59	0.14	39.6	26.53	18.38	83.06	34.38	1.79	6.03	0.88	10.28
GSB-011-22	13.03	8.18	2.13	0.10	42.12	5.69	0.14	83.71	33.75	1.59	6.42	0.92	10.32
GSR-049-22	13.53	7.82	2.44	0.15	39.04	27.68	19.76	83.10	34.14	1.73	6.14	0.90	10.29
GSR-049-22	13.23	8.17	2.63	0.15	37.71	26.38	17.39	84.89	34.09	1.62	6.42	0.92	10.40
GSR-071-22	12.75	9.1	2.6	0.17	43.4	14.38	16.98	91.13	34.56	1.40	7.15	0.96	10.77
GSB-011-22	13.92	8.54	2.42	0.13	30.62	5.79	3.02	93.37	35.79	1.63	6.71	0.92	10.90
GSB-001-22	12.82	7.09	2.59	0.13	36.54	3.9	2.47	71.39	31.93	1.81	5.57	0.88	9.53
GSR-049-22	12.81	8.13	2.4	0.14	42.17	25.24	14.71	81.80	33.30	1.58	6.39	0.93	10.21
GSR-113-22	12.87	8.55	2.34	0.16	45.72	24.04	20.04	86.42	33.99	1.51	6.72	0.94	10.49
GSR-071-22	12.56	8.21	2.45	0.14	46.42	9.7	12.34	80.99	32.98	1.53	6.45	0.94	10.15
GSR-113-22	13.58	8.45	2.7	0.16	39.67	25.68	18.31	90.13	35.08	1.61	6.64	0.92	10.71
GSW-087-22	14.08	8.9	2.42	0.13	78.79	5.78	18.05	98.42	36.56	1.58	6.99	0.93	11.19
GSB-002-22	13.38	7.51	2.12	0.13	28.79	3.61	-1.55	78.92	33.46	1.78	5.90	0.89	10.02
GSW-017-22	14.73	9.05	2.6	0.15	80.23	5.7	17.34	104.70	37.89	1.63	7.11	0.92	11.55
GSB-011-22	13.88	8.47	2.25	0.13	30.47	5.67	1.21	92.33	35.62	1.64	6.65	0.91	10.84
GSB-001-22	14.65	7.49	2.39	0.15	32.74	4.35	3.16	86.18	35.69	1.96	5.88	0.85	10.48
GSB-011-22	13.32	8.93	2.31	0.13	35	5.09	1.07	93.42	35.29	1.49	7.01	0.94	10.91
GSB-011-22	14.04	8.29	2.66	0.13	30.99	7.38	1.79	91.41	35.66	1.69	6.51	0.90	10.79
GSW-047-22	15.7	9.4	2.45	0.21	67.84	8.31	20.31	115.91	40.05	1.67	7.38	0.91	12.15
GSB-001-22	13.93	7.97	2.11	0.15	30.49	4.78	2.47	87.20	35.04	1.75	6.26	0.89	10.54
GSW-087-22	13.97	7.62	2.3	0.07	80.22	4.51	13.2	83.61	34.65	1.83	5.98	0.88	10.32
GSR-049-22	12.98	8.34	2.13	0.11	38.14	22.56	12.67	85.02	33.89	1.56	6.55	0.93	10.40
GSW-017-22	13.16	9.42	2.61	0.08	83.69	4.81	14.05	97.36	35.71	1.40	7.40	0.96	11.13
